# RNAi as a Foliar Spray: Efficiency and Challenges to Field Applications

**DOI:** 10.3390/ijms23126639

**Published:** 2022-06-14

**Authors:** Bao Tram L. Hoang, Stephen J. Fletcher, Christopher A. Brosnan, Amol B. Ghodke, Narelle Manzie, Neena Mitter

**Affiliations:** Centre for Horticulture Science, Queensland Alliance for Agriculture and Food Innovation, The University of Queensland, St. Lucia, QLD 4072, Australia; b.hoang@uq.edu.au (B.T.L.H.); s.fletcher@uq.edu.au (S.J.F.); c.brosnan@uq.edu.au (C.A.B.); a.ghodke@uq.edu.au (A.B.G.); n.manzie@uq.edu.au (N.M.)

**Keywords:** RNAi, SIGS, double-stranded RNA, foliar dsRNA spray, nanoparticles, plant uptake of dsRNA

## Abstract

RNA interference (RNAi) is a powerful tool that is being increasingly utilized for crop protection against viruses, fungal pathogens, and insect pests. The non-transgenic approach of spray-induced gene silencing (SIGS), which relies on spray application of double-stranded RNA (dsRNA) to induce RNAi, has come to prominence due to its safety and environmental benefits in addition to its wide host range and high target specificity. However, along with promising results in recent studies, several factors limiting SIGS RNAi efficiency have been recognized in insects and plants. While sprayed dsRNA on the plant surface can produce a robust RNAi response in some chewing insects, plant uptake and systemic movement of dsRNA is required for delivery to many other target organisms. For example, pests such as sucking insects require the presence of dsRNA in vascular tissues, while many fungal pathogens are predominately located in internal plant tissues. Investigating the mechanisms by which sprayed dsRNA enters and moves through plant tissues and understanding the barriers that may hinder this process are essential for developing efficient ways to deliver dsRNA into plant systems. In this review, we assess current knowledge of the plant foliar and cellular uptake of dsRNA molecules. We will also identify major barriers to uptake, including leaf morphological features as well as environmental factors, and address methods to overcome these barriers.

## 1. Introduction

First discovered in plants, a natural regulatory mechanism known as RNA interference (RNAi) or post-transcriptional gene silencing (PTGS) has been intensively studied for its role in various developmental processes, responses to stress stimuli, and antiviral defense in many eukaryotes [1,2,3,4,5,6]. This mechanism in plants utilizes Dicer-like proteins (DCLs) to process endogenously expressed or exogenously introduced double-stranded RNAs (dsRNAs) into small interfering RNA (siRNA) duplexes [7]. These siRNA duplexes are then incorporated onto ARGONAUTE proteins (AGOs), and the passenger strand is degraded [8]. The multiprotein complex guided by AGOs will form RISC (RNA-induced silencing complex) with the remaining guide strand. The RISC will bind to and cleave complementary transcripts, thereby downregulating gene expression.

Since its discovery, the potential of RNAi to facilitate resistance against viruses, viroids, nematodes, insect pests, and fungi in plants has been intensively investigated [9,10,11,12,13,14]. Host-induced gene silencing (HIGS) has been an effective RNAi approach in some crop species and involves transformation of dsRNA expressing gene cassettes into plants, followed by propagation. However, the lack of transformation protocols for many key crop species as well as exceedingly high costs, long development timelines and, frequently, issues associated with public acceptance of GMOs has led to significant interest in non-GM exogenous approaches [15,16]. Exogenous application can effectively trigger the RNAi pathway by delivering dsRNA molecules onto plant surfaces or internal tissues, which in turn target vital genes of feeding insect pests, and viral and fungal pathogens. Application techniques for delivery include foliar application, seed treatment, injection in woody plants, and absorption by plant cuttings or roots [17,18,19,20,21]. With the advances in dsRNA production systems, the cost of dsRNA synthesis has been noticeably reduced from US$12,000/g to less than US$0.5/g [22,23,24]. Thus, for large-scale protection in many broadacre and horticultural crops, foliar spray application is one of the most effective dsRNA delivery methods in terms of cost, time consumption, and labor intensity. Nevertheless, many questions remain regarding the deployment of SIGS as a viable next-generation crop protection system, notably whether intact plants in field environments can take up sufficient sprayed dsRNA for efficacy, and whether dsRNA that is taken up can move systemically to impact hard-to-reach pests and pathogens.

**Figure 1:**
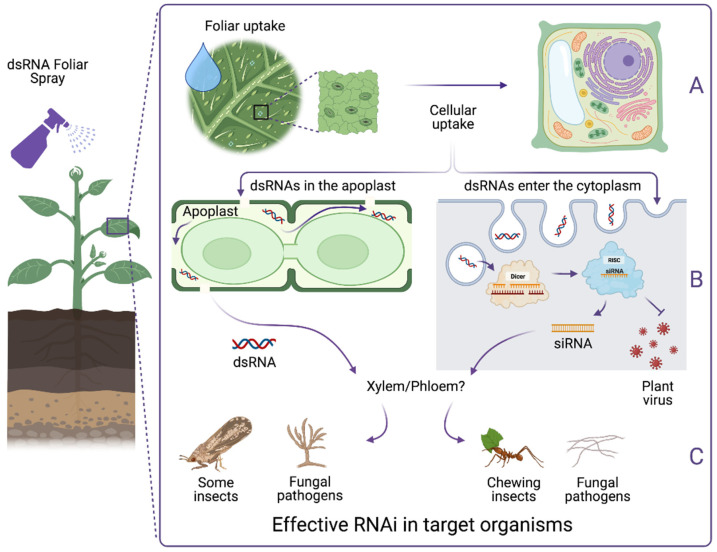
Functional crop protection via a foliar dsRNA spray to induce RNAi. (**A**)**.** Plant uptake of dsRNA can be divided into two stages: foliar uptake where sprayed dsRNA molecules from the leaf surfaces enter the interior of the leaf tissue, and cellular uptake where dsRNA molecules get taken up into plant cells. Following foliar uptake, sprayed dsRNAs may diffuse through the leaf interior and cellular uptake may occur. (**B**)**.** Once dsRNA penetrates the cell wall pores and cell membrane to enter the cytoplasm, the plant RNAi machinery can process dsRNAs into siRNAs. Produced siRNAs can lead to degradation of viral transcripts in local cells and also be transported to adjacent cells. siRNAs are likely to participate in long distance signaling through vascular bundles to other parts of the plant. It is uncertain how non-processed dsRNA in the apoplastic pathway are translocated systemically. (**C**). dsRNA/siRNAs from the plant surfaces or in the plant system can be taken up by different targets and trigger an RNAi response depending on their sensitivity to dsRNA or siRNA. Figure created with BioRender.com.

RNAi efficiency depends on the delivery of sufficient dsRNA or siRNA molecules to target pests/pathogens (Figure 1). In many crop protection scenarios, topically applied intact dsRNA needs to be internalized and systematically transported to distal plant tissues (Figure 1A,B). Fates for internalized dsRNA include cellular uptake and processing into siRNAs (which is desirable for protection against viruses), movement to vascular tissues and systemic transport as intact molecules, or degradation. Potent RNAi effects in insects could be achieved by ingesting long dsRNAs or in some cases siRNAs [25,26,27,28]. However, protection from insect pests is a multifaceted scenario whereby the dietary RNAi response can be affected by feeding behavior, life stage and the insect’s preferred attack point on the plant [29,30]. Chewing insects are an ideal candidate for SIGS, as they can easily take up a large amount of topically applied dsRNA by feeding on leaves. Plant uptake of dsRNA provides few benefits in this scenario. In contrast, RNAi-based protection from sap sucking pests predominantly relies on dsRNA uptake into and movement through the host plant’s vascular tissues (Figure 1C). Eggs laid on the plant can be challenging to target due to hard shells and relatively little active interaction with dsRNA-treated plant surfaces [31]. Insects such as stem borers may not receive a sufficient dsRNA dose due to reliance on high cellulose content food rather than live tissues and, additionally, they can also disrupt the plant’s dsRNA transport system. In some circumstances, dsRNAs can be sprayed directly on insect pests residing on plants, as dsRNAs can penetrate the cuticle of species such as *Ostrinia furnacalis*, *Acyrthosiphon pisum* and *Diaphorina citri* [32,33,34]. However, the traditional cuticle penetration route for dsRNA application is not applicable to all insect pests; for example, in insects with thick cuticles like coleopteran insects [35], or insects preferring to hide in plant parts where spray application is not possible.

A critical characteristic of the RNAi mechanism in plants is the self-amplifying gene silencing effect facilitated by RNA dependent RNA polymerase (RdRp) enzymes. Unfortunately, active RdRps are generally not found in some agricultural pests such as insects but are found in others like fungi [36]. In addition, although siRNAs can trigger the RNAi effect in some insects, it has been reported that the environmental RNAi response in others such as western corn rootworm (WCR) could only effectively be triggered by dsRNAs longer than 50 bp in length [27,37,38]. This suggests that unprocessed exogenous dsRNAs inside the plant system could be essential for successful RNAi-based biocontrol in many insect pests (Figure 1B,C). A notable example includes, but is not limited to, the western corn rootworm larvae and adults’ indifference to siRNA in transgenic RNAi maize [21]. However, sustainable dsRNA production through chloroplast transformation to overcome the processing issue could provide sufficient unprocessed dsRNA effector molecules to effectively control various insect pests in major crops such as WCR, cotton bollworm, and Colorado potato beetle [39,40,41]. As well as the previously mentioned public acceptability and crop transformation issues associated with GM technology, application of exogenous dsRNA may also circumvent insect efficacy limitations associated with dsRNA processing in plant cells, provided cellular uptake is minimal.

Recent studies have confirmed the potential of RNAi in inhibiting fungal growth and pathogenicity through the cross-kingdom RNAi and environmental RNAi [42,43]. For example, siRNA duplexes and long dsRNAs from the plant surfaces can be taken up by *Botrytis cinerea* and *Fusarium* species and effectively inhibit fungal growth [20,42,44,45]. Several studies have also described vesicle mediated transport of small RNAs between host plants and fungal pathogens [43,46,47]. However, some fungal pathogens such as *Zymoseptoria tritici* and *Colletotrichum gloeosporioides* appear recalcitrant to exogenous RNAi even though they have functional RNAi pathways, due to the inability to take up dsRNAs or siRNAs. Additionally, some fungal species lack key RNAi components altogether, as observed in examples such as *Ustilago maydis* and *Saccharomyces cerevisiae,* which are undesirable targets for RNAi-mediated control [48].

Despite extensive studies on the RNAi effect via SIGS on various pests and pathogens [20,22,28,49,50,51,52,53], the mechanisms for foliar dsRNA uptake and subsequent entry into cells are yet to be fully recognized. Understanding these mechanisms is however crucial for development and optimization of RNAi-based crop protection at scale. For a significant RNAi response and effective control of pests and pathogens, sprayed dsRNAs must overcome several barriers of the leaf surface prior to uptake, then translocate to various parts of the plant for systemic protection. In some situations, such as protection against viral pathogens, cellular uptake and dsRNA processing is essential (Figure 1B).

To date, no specific path has been confirmed for foliar dsRNA uptake, though stomata have been suggested as an entry point [54]. Once internalized, dsRNA may be partially processed into small RNA duplexes by the plant’s DCLs upon traversing the plasma membrane [20]. These small RNA duplexes could then be transported via the plasmodesmata (symplastic pathway) to adjacent cells, most likely by the vascular bundles to distal tissues, or by extracellular vesicles to fungal pathogens [45,46,55,56,57]. On the other hand, if not processed or degraded, dsRNAs could remain in the apoplast and follow the apoplastic pathway to the vascular tissues for distal translocation (Figure 1B) [20,58].

Since various physiological, molecular, and environmental factors can constrain/limit the efficacy of topical RNAi, a better understanding of these limiting factors is fundamental for the application of a sustainable foliar spray in crop protection. In this review, we discuss the current knowledge of foliar and cellular uptake of dsRNAs, with an aim to identify barriers to efficient RNAi and to propose future directions for improvements of dsRNA delivery methods.

## 2. Environmental Factors as Barriers to Efficient Plant Uptake of dsRNA

**Figure 2:**
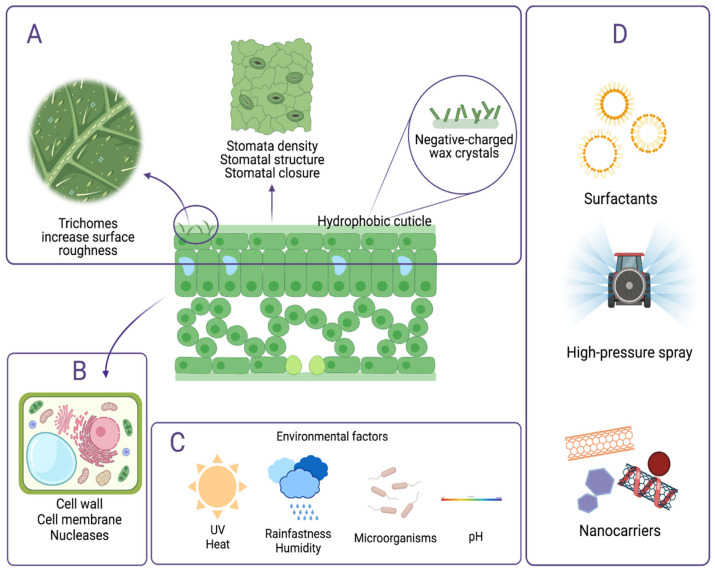
Overview of barriers to efficient foliar and cellular uptake of dsRNAs. (**A**)**.** Leaf wettability determined by trichomes, stomata, hydrophobic cuticle, and wax crystals acts as a barrier to foliar uptake of sprayed dsRNA. (**B**). Cell walls and cell membranes may hinder cellular uptake of dsRNA after sprayed dsRNA gets inside the leaf from the surface. (**C**). Environmental factors can contribute to degradation of dsRNA on the plant surfaces, thus acting as limiting factors to plant uptake of sufficient dsRNA. (**D**). Preferable procedures used to overcome these barriers and enhance uptake include the use of surfactants, high-pressure spray, and nanocarriers such as carbon dots, clay nanosheets, and single-walled carbon nanotubes. Figure created with BioRender.com.

RNAi efficacy can be impacted by the persistence and stability of topically applied dsRNAs prior to entry into the plant. Since foliar uptake of dsRNAs is not an immediate process, a longer retention time of dsRNA molecules on the leaf surface can provide a stable supply of dsRNA. The integrity of dsRNAs prior to entry into the cell is also required for binding to Dicer proteins to produce siRNAs [59]. The persistence and stability of sprayed dsRNAs can be significantly affected by environmental factors such as UV, heat and pH, which can lead to variation in RNAi responses due to the degradation of dsRNAs on the plant surfaces (Figure 2C). Additionally, biotic factors such as microorganisms could also reduce the half-life of dsRNAs via nuclease activities [60,61].

Although RNA is often viewed as inherently unstable in the environment, reports have demonstrated higher UV-resistance for RNA compared to DNA [62]. However, a loss of biological activity has been observed for dsRNA after exposure to UV light for as little as an hour, likely due to degradation [19]. Nonetheless, there has yet to be further evidence for how UV light affects dsRNA in terms of stability and RNAi efficacy in field conditions.

Another factor that may impact dsRNA degradation is pH. In many cases, the leaf surface is slightly acidic, but there is an interspecific variation in leaf surface pH [63]. This variation, which is affected by leaf physiology and ion availability in the environment, may play a role in RNAi efficiency among crop species. RNA is more stable in acidic conditions than alkaline due to its chemical composition [64]. Although dsRNA may be resistant to some degree from alkaline hydrolysis, the precise mechanisms and broad range applicability of this remains to be investigated [65]. Given the above points, preventing dsRNA degradation from alkaline hydrolysis via chemical modification or association with stabilizing nanoparticles would significantly benefit downstream RNAi applications.

Sufficient supply of dsRNA for robust RNAi is partially dependent upon retention on plant surfaces under irrigation or rainfall. Sprayed dsRNA on potato leaves has been shown to be persistent and, once dry, was not significantly washed off [19]. In contrast, a later study using fluorescent dye and confocal microscopy revealed that naked dsRNA but not dsRNA incorporated into BioClay-LDH (layered double hydroxide) complex was readily washed off tobacco leaves [53]. Whether this contradictory set of results is due to different washing methods or different leaf morphologies, utilization of surfactants or nanoparticles such as LDH in dsRNA foliar sprays may reduce or prevent wash-off by irrigation or rainfall. Another increasingly used method for field spray is the use of drone (unmanned aerial vehicle (UAV)) systems, which take advantage of the airflows to deliver a large amount of the treatment agent on the abaxial surface of the leaves [66]. The implementation of UAVs has gained popularity in smart crop monitoring and pesticides management as it can provide precise monitoring, large area coverage, timely operation, and optimized operation parameters, which in turn can further improve the effectiveness of pesticide application [67,68]. However, this has not yet been tested for foliar spray of RNAi under field conditions.

Candidate dsRNA foliar uptake pathways and the physical barriers to uptake of dsRNA molecules sprayed on the leaf must pass several hurdles to enter the plant system. These include morphological leaf features and their properties, as well as the way leaf parts interact with the environment, and natural secretion (Figure 2).

### 2.1. Leaf Wettability

Plant surfaces are crucial for defense against various biotic and abiotic stress factors [69]. Plant leaf surface morphology also contributes to leaf wettability, which is the ability to retain moisture from dew, rainfall, fog, or irrigation. Low leaf wettability is advantageous and helps prevent disease occurrence. The frequent presence of water on leaf surfaces provides favorable conditions for insects and fungal growth, making plants more susceptible to diseases. Furthermore, high water repellence facilitates cleansing of foreign particles like dust or pollutants on the leaf surface, preventing them from increasing leaf surface temperature and inhibiting stomatal closure [70]. Characteristics including the cuticle, cuticular wax, and trichomes play a key role in determining the leaf surface wettability (Figure 2A) [71,72,73,74]. The presence of trichomes increases the roughness of the leaf surface, which lowers leaf wettability (increased hydrophobicity), and thus lowers foliar water uptake [75,76]. As dsRNA molecules are applied as an aqueous foliar spray, leaf wettability plays a critical role in the deposition of dsRNAs within the spray droplets on the leaf surface, and likewise, for the foliar penetration of sprayed dsRNAs into the plant. As leaf wettability decreases, dsRNA as a topical spray will be more likely to bead and roll off the leaf surface, with a lower likelihood of penetrating the surface to enter the leaf interior. Hence, leaf wettability is the first barrier to foliar dsRNA uptake that needs to be considered for successful SIGS.

### 2.2. Cuticle and Wax

Besides the cuticle’s role in downregulating cuticular transpiration, it also limits foliar uptake of pesticides, herbicides, nutrients, and growth factors [77,78]. The structure and composition of the cuticle may vary widely among plant species, but the thickness of the cuticle typically stays between 1 and 10 μm [79,80]. Generally, the cuticle consists of an insoluble polymer cutin matrix embedded with wax. Wax, which is composed of nonpolar soluble lipids, deposits on the cuticular surface (epicuticular wax) or within the cutin matrix (intracuticular wax) [77]. In addition to regulating water loss by increasing resistance to vapor flow, epicuticular wax is also responsible for creating a hydrophobic layer repelling water from leaf surfaces [81]. Another notable characteristic of epicuticular wax is the varying shapes of wax crystals among plant species under different environmental conditions. Examples include the plate forms observed in citrus species, rodlets in *Picea* and *Gingko*, and granular forms in *Eucalyptus* [82]. The shape diversity, as well as the thickness of epicuticular wax on crops such as wheat, can act as an enhanced physical barrier to foliar uptake of water and solutes, with the likelihood of also restricting foliar uptake of sprayed dsRNAs.

The permeability of cuticles varies among plant species and developmental stages [78]. Generally, water and solute permeabilities of cuticular wax increase as temperature increases and as the size of organic solutes decrease [78]. Despite the assumption that plasmodesmata are limited to the interior of plant tissues, plasmodesmata have also been identified in the outer walls of epidermal cells [83]. These structures, called ectodesmata, establish a passage for the transport of external substances to the interior of tissues. Foliar water uptake (FWU) has been suggested to be a direct diffusion of water through the cuticle via cuticular aqueous pores and ectodesmata due to the presence of hydrophilic phenolic compounds, polysaccharides, mucilage cells in the mesophyll, and a water potential gradient [73,77,83,84,85]. However, in the leaf, ectodesmata remain covered by the cuticle, suggesting uptake by this pathway will still be impeded by many physical barriers. Alternatively, foliar water uptake could also occur through leaf structures such as the stomata aperture, guard cells, trichomes, hydathodes, or epistomatal mucilage plugs [72,86,87,88,89]. Currently, no conclusive data indicates whether dsRNA molecules could be taken up into the plant via aqueous pores due to size restriction (~1 nm) [90]. Hence, it is reasonable to presume that sprayed dsRNAs with a minimum of 3.2 nm in diameter and 100 nm in length (~300 bp) would primarily enter the plant through larger openings such as the stomata, the ectodesmata, or the hydathodes [59].

### 2.3. Stomatal Aperture

Stomatal aperture regulates transpiration and gas exchange in terrestrial plants. Due to their importance to plant survival and growth, stomata have been studied extensively. Initially, it was hypothesized that infiltration of foliar-applied solutions occurred by mass flow through stomata openings [86,87,91,92]. However, stomata flooding could restrict gas exchange and hinder photosynthesis, making spontaneous infiltration of aqueous solutions unlikely. Instead, solute transport through the stomata was shown to be independent of aqueous solvent penetration [86,93]. It was demonstrated that the stomata allowed entry of solutes along the surface of guard cells, which are also subjected to cuticular surface wettability constrains via trichomes and wax, which can keep solutes away from the guard cells [86,93,94,95]. This uptake process, though not applicable in all stomata, was suggested to be facilitated by “reverse transpiration”, in which water vapor diffuses through the stomata, bringing solutes into the leaf; hence the occurrence followed by the evaporation of water films on the leaf surface and at the stomatal pore is necessary for foliar uptake of dsRNA, should it follow this pathway to enter the leaf via water vapor [96,97,98,99]. It should also be noted that stomata distribution is species-dependent and environment-dependent. In many plant species, specialized structures derived from stomata, known as hydathodes, are unregulated openings usually found in the epidermis or leaf margin. Besides their primary function in guttation, they were reported to be involved in the absorption of leaf surface water [72]. Nonetheless, the lack of evidence whether this finding applies to other plant species suggests that stomata opening is likely the main path through which dsRNA can enter the leaf interior. In an agricultural setting where spray application in the field is done from above, the absence of stomata on the adaxial surface can also act as a barrier to foliar uptake of sprayed dsRNAs.

## 3. Possible Methods to Overcome Barriers to Foliar Uptake of dsRNA

Due to stomatal anatomical and physiochemical features, uptake of foliar-applied dsRNAs into the leaf interior is restricted. Potential methods have been developed to achieve robust RNAi responses by increasing leaf wettability, enhancing cuticle penetration, and boosting solute transport. These methods include disrupting the cuticle structure by abrasion, utilizing high pressure or surface-active agents (surfactants), or chemically modifying stomatal aperture (Figure 2D) [87,93,99,100,101].

Surfactants in combination with chemical pesticides have been widely used in plant disease management [102,103,104,105]. Surfactants are usually added as adjuvants to lower the interfacial tension between leaf surfaces and liquids and enhance spreading, thus allowing pesticides to make contact with pest targets not easily accessible with overhead sprays [106,107]. Additionally, surfactants also lengthen the retention time of chemical sprays on plant surfaces and increase penetration for absorption into the plants or pests [103,106]. However, risks should be carefully evaluated to avoid damage to the plants, the environment, or off-target organisms [108,109,110].

High-pressure spraying of siRNAs was reported to have induced both local and systemic silencing of the GFP transgene in *Nicotiana benthamiana* where mere spraying, syringe injection, and infiltration of siRNAs failed to induce RNA silencing [111]. In contrast to this result, a more recent study claimed that no silencing of GFP genes in *N. benthamiana* was observed upon the high-pressure spray of dsRNAs [112]. This study suggested that the inadequate uptake of dsRNA into the plant cells led to insufficient siRNA production by the plant’s RNAi machinery, resulting in an unsatisfactory RNAi effect on endogenous genes. This implies that while dsRNAs may be internalized into the leaf, dsRNAs do not necessarily enter plant cells. Thus, barriers to cellular uptake of sprayed dsRNA following foliar uptake should also be considered.

## 4. In Planta Transport of Sprayed dsRNA Molecules following Foliar Uptake

Systemic spreading of RNA silencing via the phloem has been reported in studies using plant transformation [113,114]. When sprayed dsRNAs are internalized into the leaf and diffuse through the epidermis and then the mesophyll, there are two possible scenarios: (i) dsRNA molecules permeate the cell wall then the plasma membrane and may or may not be processed into small RNAs in the cytoplasm, or (ii) dsRNAs are not taken up into the cytoplasm and are translocated short distances or long distances throughout the plant as unprocessed molecules by other means. It was suggested that upon traversing the plasma membrane, dsRNA would be partially processed by the plant’s RNAi machinery into small RNAs [115]. These small RNAs would most likely be transported through the plasmodesmata to adjacent cells, then to the phloem and ultimately long-distance to other parts of the plants [56,116]. Alternatively, dsRNAs not entering the cytoplasm would diffuse through the apoplast to the vasculature for long-distance transport [18,117]. The possibility that small RNAs are incorporated into extracellular vesicles has also been proposed for cell-to-cell communication and plant–fungi interactions [43].

A study with petiole absorption or trunk injection of dsRNA showed fluorescence signals accumulating exclusively in the xylem of the apple plant (*Malus domestica*) [18,58]. This study speculated that RNA molecules (most likely dsRNA) may have been too large to be transported into phloem cells. However, it should also be noted that petiole absorption of dsRNA in this study could have resulted in the accumulation of dsRNA signal in the xylem [118]. In contrast to this statement, other studies have successfully sequenced siRNAs from phloem sap while xylem was found to be RNA-free [119,120,121]. Long-distance translocation of exogenously applied dsRNAs has been reported where strong resistance against Fusarium was observed in non-sprayed distal parts of detached barley leaves [20]. This study presented data suggesting that movement of dsRNAs in the vascular system occurs in the source-to-sink direction, thus making an argument that phloem is a participating pathway for translocation of dsRNAs. The authors postulated that exogenous dsRNAs first entered the apoplast, and then translocated to the xylem, then to the phloem through an as yet unknown mechanism. This postulation could not be ruled out as xylem-to-phloem exchange exists together with the exo/endocytosis mechanism of materials from the apoplast to the xylem vessels [118,122,123,124]. Another study showed gene knockdown in phloem-feeding aphids, which indicated delivery of unprocessed dsRNA from the vascular tissues to the insect target [117]. Whether long dsRNA could be transported in the phloem is important considering many plant pests are phloem-feeding insects, and many of them have been reported to be more sensitive to RNAi from feeding on long dsRNAs compared to feeding on small RNAs [27,38]. That bidirectional flow of dsRNA, which is primarily considered to associate with phloem transport, is also crucial for translocation of dsRNAs to target pathogens at the plant stems and roots. Systemic transport of spray-applied dsRNA in plants is still actively being investigated as the underlying mechanisms of transport and activity are still largely not understood.

## 5. Barriers to Cellular Uptake of dsRNA

Under a scenario where sprayed dsRNAs bypass the numerous physical barriers and are internalized into the leaf interior, dsRNA molecules still face more barriers to entering the plant cells (Figure 2). All plant cells are enveloped in a cellulose wall formed of several intertwining biopolymers for support and resistance to the turgor pressure of the plant protoplast. The porous cell wall acts as a non-specific barrier allowing passage of molecular and ionic components from adjacent cells or the extracellular environment to the plasma membrane [125]. Due to its selectivity principle, the plasma membrane is a major barrier for cellular uptake of sprayed dsRNAs.

It has been suggested that cell wall porosity may be subjected to changes depending on development stage and cellular responses to the environment [126]. Though wall porosity restricts the size of molecules that can permeate the cell wall, generally, macromolecules up to 10 nm can penetrate the cell wall, whereas the transport of dsRNA requires a minimal pore diameter of 3.2 nm [59,127]. A recent study has suggested that the limiting size for double stranded DNA (dsDNA) cellular uptake is between 50 and 90 bp [128]. However, it should be noted that this claim was based on measuring the quantity of dsDNAs present in stimulated endosomes at the time. This could have been due to degradation of dsDNA following endocytosis or because the mechanical properties of dsDNA, while remaining sequence-dependent, similar to dsRNA, are strikingly different from those of dsRNA [129]. Furthermore, several studies have demonstrated exogenous dsRNA-mediated protection against plant viruses. This means that the exogenously applied dsRNAs were successfully taken up into the plant cells and then processed into siRNAs to inhibit viral infections [53,130,131,132].

To gain access to the plant RNAi machinery for small RNA production, dsRNAs will need to enter the cytoplasm. Due to being made up of different components to the cell wall, the plasma membrane is a highly selective barrier restricting the entry of extracellular particles. The cell membrane is a negatively charged lipid bilayer containing trans-membrane channels and transporters. These channels and transporters regulate active transport, osmosis, and diffusion of small molecular weight materials across membranes, but their roles in dsRNAs uptake remain unclear. On the other hand, engulfment by endosomes could represent a major entry point of extracellular particles. However, the mechanism triggering the endocytosis of dsRNA remains to be unidentified, making it a challenge to further investigate whether topically applied dsRNA can be taken up by plant cells via this mechanism. Non-stimulated endosomes were reportedly unresponsive to the internalized dsDNA; thus, inducing endocytosis with transfecting reagents or incorporation with carbon-nanocarriers may facilitate efficient small RNA production in sprayed plants [128,133].

## 6. Nanocarriers as an Effective dsRNA Delivery Method

To tackle various physical and biochemical barriers as well as provide protection, nanoparticles, with sizes ranging from 1 to 500 nm, have been employed in topical dsRNA delivery in plants (Figure 2D) [52,53,134,135,136,137,138]. Positively charged nanoparticles, including but not limited to metals or cationic polymers, are designed to bind to dsRNA, forming biodegradable complexes for sustained release of dsRNA over time. Studies have revealed that formulation with nanocarriers could protect dsRNA from UV and nuclease degradation [53,139]. The use of nanoparticles has also demonstrated increased persistence of sprayed dsRNA on leaf surfaces after rinsing [52,53]. These approaches showed that dsRNAs in complex with layered double hydroxide clay nanosheets (BioClay) largely remained on leaves, while unprotected dsRNAs were readily washed off. Another valuable aspect of nanoparticles is the potential to improve foliar and cellular uptake of sprayed dsRNA [136,137,140,141]. dsRNA-nanoparticle complexes are overall positively charged, and thus can enhance penetration through the negatively charged plasma membrane [142]. Delivery of siRNAs with carbon dots enhanced cellular uptake even with low-pressure spray application, indicated by significant silencing of the plant’s endogenous genes [137]. In addition, single-walled carbon nanotubes (SWNTs) have been investigated for the capability to deliver DNAs and siRNAs into intact plant cells. Cellular uptake of SWNT/DNA conjugates demonstrated the potential of utilizing SWNT as nano transporters to different plant cell organelles [140]. More recently, promising results showed that SWNTs could protect siRNAs from nuclease activities and efficiently deliver DNA and siRNAs to the cytoplasm, triggering endogenous gene knockdown [135]. Though studies on the delivery of dsRNA with nanocarriers are still limited, these results have shown that further improvements of nano-delivery methods could prove important to practical field application of topical RNAi as a spray.

## 7. Concluding Remarks

Physical and biochemical barriers notably limit the entry of topically applied exogenous dsRNA into the plant leaf tissues. These barriers provide possible explanations for the inconsistency of the topical RNAi effect among different plant species, targets, and the environmental settings [128,143]. The interspecific variation in leaf morphologies also indicates the need for host-dependent dsRNA delivery methods, requiring case-by-case evaluation of uptake efficiency upon selection of plant host and pathogen targets for topical RNAi. Several efforts have been made to enhance foliar dsRNA uptake including cuticle abrasion, high-pressure spray, surfactants, and association with nanoparticles. Although highly effective, formulation with nanoparticles must be designed case-by-case, depending on the target species, and to avoid environmental risks [142]. Furthermore, the use of nanoparticles could also enhance dsRNA persistence on plants and provide dsRNA stability in an uncontrolled environment. Movement of dsRNA within the plant and cellular uptake of sprayed dsRNA can be crucial for targeting specific plant diseases and conferring systemic protection. Thus, conducting additional studies on incorporating these methods into the designs for dsRNA delivery as a foliar spray is essential for the feasibility of sustainable crop protection with RNAi in the wide field.

## Data Availability

Not Applicable.
